# Integrating Urban Land Tenure Security in Health Determinants: The Design of Indicators for Measuring Land Tenure Security and Health Relationships in Developing Country Contexts

**DOI:** 10.3390/ijerph19053080

**Published:** 2022-03-05

**Authors:** Walter Dachaga, Walter Timo de Vries

**Affiliations:** Chair of Land Management, Department of Aerospace and Geodesy, School of Engineering and Design, Technical University of Munich (TUM), 80333 Munich, Germany; wt.de-vries@tum.de

**Keywords:** indicators, land tenure, urban health, global health, health, tenure security, land use, social determinants of health, land management, informality, urban neighborhoods

## Abstract

Both urban land tenure insecurity and poor urban health outcomes are research topics of urban geographers and health experts. However, health outcomes or patterns are hardly measured in relation to land tenure security. There are no clear measures or indicators of if and how these two issues interrelate and which type of land tenure deficiency is likely to lead to which kind of health outcomes or patterns. To address this knowledge quandary, we reviewed literature to identify which characteristics of land tenure could relate to which types of health outcomes. The review found four specific land tenure security pathways which significantly influence health outcomes. For each of these, it is possible to identify a set of indicators which could measure the extent of interrelation between land tenure security and health. The result of this process is the design of a list of 46 land tenure-enabled indicators that can be applied empirically. The indicators demonstrate how to design a transdisciplinary approach that connects land management and global urban health knowledge spaces.

## 1. Introduction

Understanding persistent and increasing spatial inequalities in health remains an important objective across different fields of academic enquiry for geography, epidemiology, and public health [1]. Seeking explanations for the spatial divide in health is however crucial to offer relevant and timely insights for improving health outcomes particularly among disadvantaged groups. A key driver for the increasing geographical differences in health is the disparity in the physical environment, which can either sustain or damage health outcomes [1]. This notion recognizes that unequal distribution of resources and opportunities as well as an uneven access to good quality physical environments are likely to account for social disparities in health outcomes.

The framework of social determinants of health has been powerful to understand why and where health outcomes vary conceptually. Instead of focusing only on the direct causes of pathology and disease, this framework incorporates pathways of behavior, environment, and resources [2]. It has also birthed the idea that context matters for individual health and, thus, provides a justification to investigate the role of different types of contextual factors in the production and maintenance of health variation. Put explicitly by Connoly [3], there is a growing academic and policy interest in connecting challenges of a majority urbanized world to questions of health and disease. Consequently, urban geographers re-engage with the idea that place contributes to health variations, as it contains social relations and physical resources that affect health. Although the place and health conundrum mostly concentrate on the hard aspects of place such as landscapes, housing, and housing conditions and neglects the soft aspects of place, it is also the case that the soft (intangible) aspects of place such as land tenure (security) shape the hard parts of place that influence variations in health outcomes.

In informal urban areas, the simultaneous manifestation of increased health and disease burdens (i.e., the total, cumulative consequences of a defined disease or a range of harmful diseases with respect to disabilities in a community [4]) and increased land tenure insecurity beg the question if variations in land tenure security and variations in health outcomes relate to each other. This relational puzzle currently draws on descriptive and spatial research on where informal settlements arise and how informal settlements manifest inadequate access to safe water, sanitation and other infrastructure, poor structural quality of housing, overcrowding, and insecure residential status, which portrays informal settlements as areas of not only deprivation and land tenure insecurity, but as locations of poor health outcomes and health inequities [5].

Providing land tenure security is a well-known and widely applied strategy of development agencies to improve livelihood, well-being, and quality of life [6,7,8,9,10,11,12,13]. Land tenure security and land use policies can influence population health by supporting or stymieing opportunities for employment, housing security, political participation, education, protection from environmental risks, access to primary health care, and a host of other social and physical determinants of well-being [14]. This suggests that enhancing land tenure security is either directly or indirectly related to health and well-being outcomes [15,16,17,18,19]. Despite the supposed link between land tenure security and health, measuring how much which aspects of land tenure security contribute to which aspects of health is a major unknown and therefore one of the challenges to promoting more healthy and equitable cities.

Whereas there exists a multitude of indicators for measuring land tenure security in contemporary literature [20,21,22,23,24], none of these indicators to the best of our knowledge explicitly incorporates variables of health—a necessity for measuring the linkages between land tenure security and health. This is partly because there is a dichotomy on the conceptualization of what constitutes land tenure security and hence its measurement. While some scholars posit that having a title to land is direct evidence of having land tenure security [25,26,27], others claim that the individual’s perception of tenure is a better measure of land tenure security [21,28,29,30,31,32,33]—the basic law of social sciences states that people act upon what they believe is true and valid. We assume in this research that both claims are valid, but that there are also several other factors which must be factored into measuring a person’s level of absolute and relative land tenure security. Focusing on legal tenure or perception only, obscures the plethora of factors that either increase or decrease perceived land tenure security which, according to many scholars [21,25,31], is the driver of investment and not land titles. These dynamics of land tenure security are well noted across research in Africa, Asia, and other developing country contexts, which suggests alternative sources of tenure security rather than land titles only [30,31,34,35,36,37,38].

A person may perceive land tenure security irrespective of a title, and vice versa. Yet, other factors such as living in a planned area, living in a disaster-prone area, political affiliation, experiencing previous contestation of tenure, or living in a neighborhood with certain class of people are equally important in assessing land tenure security. Therefore, measuring land tenure security should aim at deriving a composite index based on an aggregation of several indicators that influences a person’s level of land tenure security.

Whereas in the land management domain the issue of land tenure security is still an open debate, the health knowledge domain has multiple sets of accepted metrics to measure single pathogenic exposures or risk factors. Yet, these measures often ignore both community assets that promote health equity and the cumulative impacts on health from exposure to multiple urban environmental, economic, and social stressors such as land tenure security [14]. Therefore, land tenure security is not included in the possible range of social policy tools to improve health outcomes. Consequently, indicators are lacking and needed, especially in urban settings, that measure the link between land tenure security and health.

Six key criticisms can be made about existing scholarship on land tenure security and health. First, there is paucity of literature that explicitly measures the relationship between land tenure security and health. Second, most studies have considered single land tenure security attributes (e.g., possession of title or ownership versus rental housing) without empirical investigations that consider a range of land tenure security features. Including multiple features of land tenure security will provide richer insights into the nuances of land tenure security that influence health outcomes and inequalities. Third, attempted investigations on land tenure security and health hardly demonstrate the mechanisms and/or pathways of association between land tenure security and health. Fourth, land tenure security and health inequalities have largely remained separated domains in academic research circles. Fifth, despite existing connections between land tenure security and health, there is little progress in measuring this connection with comprehensive indicators. Finally, existing indicators of land tenure security and health exist in their separate domains and have never been combined to measure the relation between land tenure security and health. These omissions in literature pose challenges in global urban health and land management research and have prevented the establishment of a knowledge base on which public policy on social determinants of health could be premised. This study responds to these literature omissions by building upon a previous conceptualization of land tenure security and health nexus [15]. It proposes composite indicators that measure the pathways through which land tenure security and health are linked, and that project if and how land tenure security policies can act as a potential intervention to promote good health or an enabler of poor health where it is lacking. This study is therefore a design (of a data collection and data analysis methodology) research article in which we introduce a data collection framework in the form of indicators.

By identifying and intersecting variables and indicators of land tenure security and health, we unearth the land tenure security induced health indicators that can influence the health profiles of individuals and households. In the next section, we posit land tenure security as preventive medicine based on existing theoretical and conceptual propositions on the linkages between land tenure security and health. Thereafter, we present a synopsis of land tenure security and health indicators development in Section 3, followed by materials and methods in Section 4. In Section 5, we frame a set of indicators for measuring the land tenure security and health nexus. Finally, we discuss and conclude the study in Section 6.

## 2. Land Tenure Security as Preventive Medicine: A Conceptual Approach to Health

A broad body of scholarship shows that ensuring land tenure security by establishing rights and effectively enforcing and adjudicating those rights commonly has positive impacts on livelihoods and living conditions, by reducing landholders’ uncertainty and supporting investment in development [6,7,30,39,40,41,42,43,44,45,46,47]. Thus, access to secure land is a precondition for securing basic living conditions, livelihood opportunities, and a means to poverty reduction. However, does secure tenure specifically promote health or does the lack of it facilitate poor health outcomes? The evidence on this land tenure security and health link is mixed, with some researchers documenting a significant relationship between land tenure security and health [16,18,48,49,50,51], while others report little to no effect of land tenure security on health [52,53]. Various and sometimes contrasting propositions exist to describe and detail how, where, and under which conditions land tenure security and health relate. Some suggest that high income or superior psychological characteristics may facilitate more secure land tenure [54]. Thus, income and psychological characteristics predict health independently without secure land tenure. Others take a reverse causality stance, arguing that health is a predictor of land tenure security [55]. However, we posit that land tenure security is an important promoter of health due to its role in shaping socio-physical and environmental conditions in which people live. Land tenure security is a proxy for both economic status and psychological characteristics that affect health outcomes. Although land tenure security is not an actual cure, one could describe it as a preventive medicine because it enables resilient conditions for people to live healthy lives and promote good health. Conversely, land tenure insecurity increases the vulnerability of people to poor health and well-being [13]. While health outcomes in informal settlements can be attributed to poverty, land tenure security can be counter-argued as a poverty alleviation intervention. As such, land tenure security may act in unison with income to improve well-being and health. On the contrary, land tenure insecurity, in unison with poverty, exposes people to a double disadvantage of land tenure insecurity and poverty, which affects health. Urban poverty manifests itself in severe overcrowding, homelessness, and environmental health problems caused by the worsened state of access to shelter and security of land tenure [56]. Achieving security of land tenure has the potential to break the poverty cycle, as it is intrinsically linked on multiple levels to accessing basic urban services and investment [9]. Hence, we take on a widely assumed but largely unexplored proposition that land tenure security is a determinant of health outcomes through four paths: environmental justice, social cohesion, psychological security, and infrastructure access [15]. To emphasize how relevant enhancing land tenure security is to improving health outcomes, Corburn and Riley [5] noted that slums can only become healthier living environments if urban slum dwellers are offered secure land tenure to remain in place, not evicted or displaced forcefully without any realistic alternative. Residing in the same locality allows the urban poor to keep their existing social networks, improve the physical, social, and economic environment of urban places, and build upon the social, physical, and other investments already made. Lack of secure land tenure may be associated with health risks such as homelessness, increased poverty, and exposure to cold and environmental toxins, leading to infectious and non-communicable diseases [57]. Similarly, eviction threats may contribute to constant stress that can compromise the immune system and cause hypertension, cardiovascular disease, glucose intolerance, insulin resistance, increased susceptibility to infection and inflammation, and the death of neurons in the hippocampus and prefrontal cortex [57,58].

A prominent feature of land tenure security is constancy of stay, which enables daily routines of life to be carried out, control of one’s life, and construction of identities. Thus, this feature of land tenure security promotes both social and mental health. Land tenure security offers protection, autonomy, and prestige [59]: protection from the outside world and threats of eviction; autonomy to enjoy rights to land, exclude others, and make independent decisions to land; and prestige from an enhanced social status that accompanies secure land tenure.

In fact, security of land tenure is more of a psychological variable that consists of both the states of thinking and feeling [20,28,29,30]. Thus, despite the legal projection of land tenure security, it is a matter of the state of mind of the landholder. This makes land tenure security an antidote for mental health. Four characteristics of land tenure security that drive the connection between land tenure security and health, and posit land tenure security as preventive medicine, are that it is a psychological variable comprising both the states of thinking and feeling, it is a distributive right of justice, it provides access to life enabling resources, and it is a social relation with people and land, whereby the strength of this relationship can be linked to stress-buffering benefits that affect health outcomes. We leverage on these features to derive indicators of land tenure security that have health implications.

In relation to sanitation and hygiene, Joshi et al. [60] argued that demanding personal sanitation investment in situations of highly insecure tenure is not a realistic approach to improving sanitation and environmental health. Land tenure security provides people the safety net to invest in utility connectivity and sanitation infrastructure. Likewise, neighborhoods and individuals without land tenure security are rarely served well top-down due to undefined or precarious land tenure status, or bottom-up due to lack of incentive to invest in insecure areas. Thus, land tenure security is a moderator of health in influencing access to basic infrastructure and services for good health and well-being. Research findings of multiple authors [19,60,61,62,63,64,65] suggest that tenure insecurity presents both real and perceived barriers to sanitation provision or access in informal areas, which underscores the potentials of land tenure security to influence health outcomes.

Given the theoretical and conceptual connections between land tenure security and health that have been advanced so far, this study becomes imperative as it focuses attention on deriving methods in the form of indicators to measure the speculated land tenure security and health relationship. In the next section, we turn to issues of measuring land tenure security and health to derive comprehensive indicators that are common to land tenure security and health.

## 3. Synopsis and Gaps in Land Tenure Security and Health Indicators

The indicator approach is a commonly used measurement instrument which does not only apply to the field of land management and administration, but also in knowledge domains such as economic development, environment, sustainable development, and health [24]. An indicator provides a sign or signal that something exists—it is used to show the presence or state or main characteristics of a situation, condition, or object of analysis [66]. In relation to health, it is a qualitative, quantitative, or temporal variable that is used to measure (in)directly changes in health and health-related situations, which includes land tenure security.

There are many operational, methodological, conceptual, and institutional complexities inherent in defining indicators for land tenure studies [67], which makes empirical measurement of land tenure security a contentious issue [68]. The Millennium and Sustainable Development Goals, indicators 32 and 1.4.2 respectively, refer to the proportion of households with secure land tenure and proportion of total adult population with secure tenure rights to land, with legally recognized documentation and who perceive their rights to land as secure, by gender and by type of tenure. However, quantifying land tenure security has proved problematic, as assessing land tenure security goes beyond just looking for the presence or absence of formalized rights but understanding the factors that affect risks and perceptions of risks to land rights [68,69]. Two main components have been proposed as measures of land tenure security: documentary evidence of tenure and evidence of either de facto or perceived tenure [28]. The expert group meeting of urban indicators proposed a secure land tenure index as a proxy indicator regarding both the household itself and the contextual environment consisting of legal and cognitive elements. They include proof of secure tenure; perception at settlement level of secure land tenure; annual evictions within the past 5 years; women’s equal right to secure tenure; and existence of national and municipal provisions against forced evictions [56]. The commonly used land tenure security indicators, including possession of title to land, the duration, the transferability (alienability), excludability, and the exclusivity of land rights, are seen to be too narrow to depict the contextual aspects of land tenure security, and are often designed to address one or another specific characteristic of land tenure security [34,70,71].

Most indicators of land tenure security are developed by international organizations or NGOs involved in the land sector where land tenure security is usually translated into few indicators and packaged into evaluation tools or frameworks [24]. Mention can be made of, including but not limited to, the USAID Land Tenure and Property Right Assessment Tools—a collection of instruments designed around the Land Tenure and Property Rights Matrix that can be used by USAID missions to expand upon land tenure and property rights themes in their respective countries and determine how these contribute to or impede development programming [72]; Property Rights Index (Prindex)—a global survey that collects data on rates of property documentation and perceptions of tenure security [73]; Global Land Indicators Initiative—a collaborative and inclusive process for developing Global Land Indicators [22]; World Bank Land Governance Assessment Framework—a diagnostic instrument to assess the state of land governance at the national or sub-national level [74]; and LANDex—a global land index that puts people at the center of land data, democratizing land monitoring, and building a data ecosystem where all voices can be heard [75] and a host of land tenure security indicators by individual researchers and scholars [20,21,24,76].

In urban health studies, the development of metrics for measuring the health burden of individuals and neighborhoods in urban settings remains a challenge. Demographic Health Surveys collect a wide range of objective and self-reported data with a strong focus on indicators of fertility, reproductive health, maternal and child health, mortality, nutrition, and self-reported health behaviors among adults [5]. Often, the focus on land and tenurial factors of health is either limited or missing.

**Figure 1:**
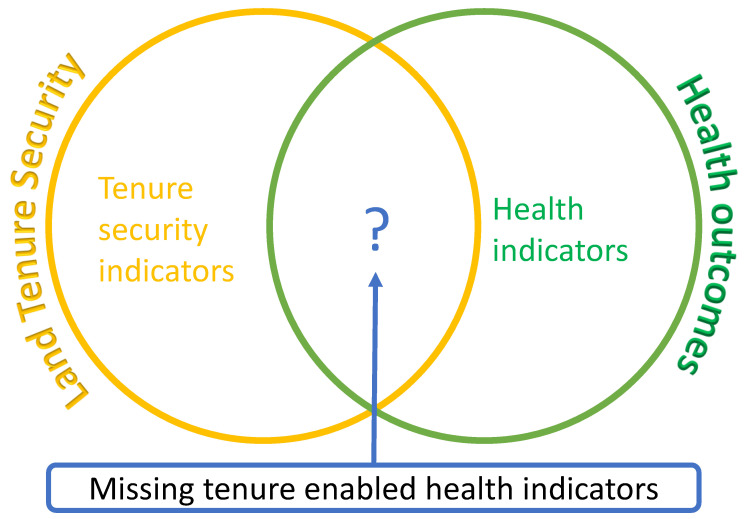
Literature gap.

Indicators have been traditionally developed in the separate domains of health and land management to independently measure health outcomes and tenure security. The few attempts to jointly measure land tenure and health were focused on the type of tenure which is also often limited to ownership and rental [77], but fails to emphasize on land tenure security and the nuances of factors that improve or threaten land tenure security. While the aim is not to develop entirely new indicators of land tenure security, this study aims at interpreting and extending the scope and indicators of land tenure security and using these as proxies to measure the connection between land tenure security and health, to the extent that these land tenure security indicators relate to some aspects of human health and well-being. In doing so, we wade away from the land tenure security measurement dichotomy of perception versus land titles, to a multifactor measure that incorporates legal documentation, circumstantial and contextual factors, and individual perceptions to ascertain an individual’s level of tenure security. We pose that perception is the best possible way to measure tenure security, but such perception is influenced by lived circumstances of tenure. The logic of this paper is to cross-fertilize variables of land tenure security with variables of health to measure the relationship between land tenure security and health in urban areas (see Figure 1).

Figure 1 illustrates that there is an intersection between land tenure security and health outcomes, and while there are separate indicators for measuring land tenure security and health, literature is missing on the set of variables and indicators that are common to both land tenure security and health. By finding indicators that are common to land tenure security and health, we would be filling a research gap in the literature.

Previous investigations of land tenure security and health linkages discussed in literature have overly simplified the measure of land tenure security and often conveyed it in simple categories of homeownership and rental housing (i.e., landlord and tenant) [56,77]. Hence, there is also the need for indicators that look beyond simple categories of owner and renter or with or without title, to contextual factors and lived experiences that have the potential of influencing an individual’s perception of tenure and subsequent implications on health. We attempt in this paper to develop a framework of land tenure security facilitated health indicators to identify and study the relationship between land tenure security and health outcomes in urban neighborhoods.

## 4. Materials and Methods

To develop indicators for measuring land tenure security and health linkages, we rely on our earlier land tenure security and health nexus conceptual framework which identifies land tenure security as related to health in four dimensions: environmental justice, psychological security, social cohesion, and infrastructure access [15]. A literature search was thus conducted to identify scales, indicators, or indices that measure tenure security, as well as indicators that measure the four identified constructs through which land tenure security and health intersect. Following a narrative approach, a review of peer-reviewed and grey literature was conducted to identify a list of tenure-based factors with the potential for a health impact.

New topics and cross-disciplinary research, such as the study under consideration, do not have enough primary research data upon which to base conclusions, which makes a narrative review appropriate for approaching both scientific and gray literature to derive new knowledge. We searched publication databases—including PubMed, Web of Science, and Google scholar—for empirical, conceptual, or theoretical evidence of the health impacts of these factors. Sample search terms included land tenure and health; tenure security and health; tenure indicators and health indicators; measures of tenure and health; land tenure and health outcomes; indicators of tenure security and health; land tenure and urban health; and urban health indicators.

Scholarship linking land tenure security and health is relatively new, and mostly talked about in land- and health-related policy briefs and reports of organizations such as the World Bank, World Health Organization, UN-Habitat, Global Land Tool Network, United States Agency for International Development, United Nations Convention to Combat Desertification, and Global Land Outlook. Therefore, we relied on such reports but also went beyond database searches to article recommendations on Research Gate, and using backward search techniques, identified, and reviewed other relevant literature. We compiled and reviewed literature that captures urban health issues, integrating aspects of land tenure, and aiming to identify key land tenure dimensions, factors, and pointers as proxies for measuring the link between land tenure security and health outcomes.

**Figure 2:**
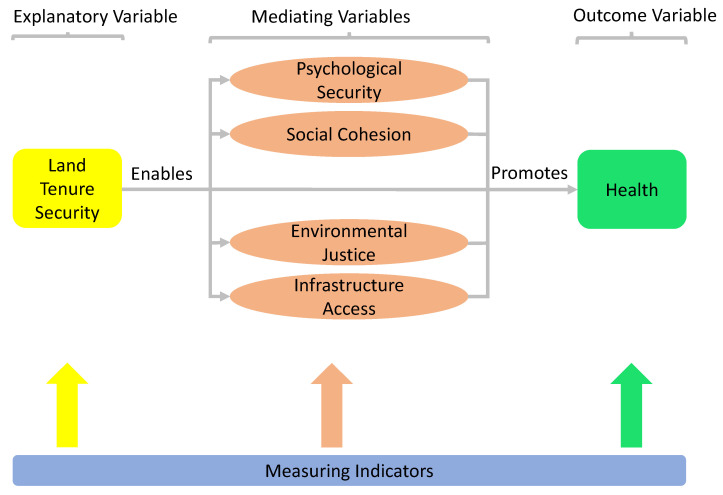
Evaluative framework for measuring land tenure security and health.

Overall, a total of 198 articles were retrieved: 173 from systematic search in research databases, 15 from article recommendations on research gate, and 10 from spider backward search strategy. From the 198 articles, 109 were excluded after reading each article’s title and abstract. The remaining 89 articles were critically reviewed to identify relevant themes and indicators of tenure security that have implications on health. Eighty of the critically reviewed articles were used in this study. Articles were included for review to the extent that they discussed aspects of land tenure security and health. Informed by propositions of the framework of social determinants of health and our previously developed conceptual framework [15], we developed an evaluative framework as shown in Figure 2 to guide the indicator development and subsequent evaluation of land tenure security and health links.

From Figure 2, the relationship between land tenure security and health is mediated by four constructs: psychological security, social cohesion, environmental justice, and infrastructure access. It follows the logic that land tenure security enables the four mediating constructs which are also known promoters of health. Therefore, tenure security also promotes health. Underlying each construct in the framework above are respective indicators that indicate the existence of each construct. First, we identified indicators for ascertaining a person’s land tenure security using a combination of subjective and objective measures of tenure security. Objective measures included those such as possession of title, duration of tenure, conflicts, and past loss of tenure or property, with subjective measures being the individual’s perceived level of land tenure security based on the objective measures of land tenure security that relate to their land tenure experiences [12]. Second, we identified pathways of land tenure security that have proven consequences for health and well-being and developed composite tenure-linked indicators to measure each of these dimensions. Each pathway constituted a mediating variable in the relationship between land tenure security and health. Hence, in measuring each variable, we were concerned with those indicators that had health salience and an association with aspects of tenure security.

We took a formative indicators approach in which indicators are viewed as causing an individual’s level or rank in the scale representing the severity of each construct, in contrast to a reflective indicators model in which observed indicators are responding to the underlying factor [2]. Thus, causality flows from the indicators to the constructs as opposed to the flow of causality from constructs to indicators, and the constructs exist because of a composition of indicators where changes in indicators can cause changes in the construct. To explore the patterns of association between tenure security and health outcomes, we examined three health measures: self-rated health, number of visits to the hospital for self-treatment, and clinically diagnosed diseases (through syndromic surveys) that represent communicable and non-communicable diseases.

Contrary to one-item measures which often shrouds the nuances and multifaceted nature of the constructs used in this study (land tenure security, psychological security, social cohesion, environmental justice, and infrastructure access), we opted for composite or multiple-item measures which enabled us to capture the different factors that constitute each construct of interest in relation to land tenure security. We considered and adapted earlier designs of indicators of land tenure security such as the Global Land Indicators and the Prindex. Currently, the most comprehensive frame of indicators to measure land tenure security is the Prindex [78]. However, these set of indicators are focused on perception of tenure, and deficient in the land tenure circumstance of people which shape their perceptions of tenure. In addition, the Prindex indicators and other existing land tenure security indicators lack a health dimension. There was the need for specific tenure security indicators that have health relevance. The novelty of the proposed set of indicators in this study is that it extends the list of Prindex indicators to include circumstantial factors that can either make people tenure secure or insecure and, in addition, demonstrates the health salience of various indicators of tenure and how land tenure security and health interrelate.

To construct the set of indicators, we followed de Vaus’ three step process of indicators development: clarifying the concepts, developing the indicators, and evaluating the indicators [79]. First, we identified and defined tenure security and health as concepts that are related and identified four constructs that help explain the relation between land tenure security and health [15]. Second, we designed indicators that help us ascertain a person’s tenure security status, and further indicators (derive from secure land tenure) that measure the constructs that link land tenure security to health. This step involved identifying existing indicators of land tenure security in literature, modifying, and interpreting them with a health lens to measure constructs that influence health outcomes. Our target was to have at least two indicators for each measured construct. Specifically, we identified indicators from existing frameworks of land tenure security and existing frameworks of urban health. Indicators from tenure security frameworks were included on the basis that they can be interpreted to affect an aspect of health, whether it be environmental, physical, social, or mental. Indicators from (urban) health frameworks were included on the basis that they can be interpreted to derive from an aspect of people to land-relations such as periodicity, assurance, provability, space, recognition, rights, privileges, restrictions, legitimacy, and livability. Next, we brainstormed and defined dimensions for classifying the indicators—these dimensions being dimensions of tenure security that are known to influence health or determinants of health which can be derived from secure land tenure. Four dimensions that were identified are environmental justice, psychological security, social cohesion, and infrastructure access. Each indicator was classified under one of these four categories and indicators that did not fit any category were eliminated. Third, we evaluated the indicators to make sure that they could measure the concepts they are designed to measure (validity) and that we could rely on answers the respondents would provide. To achieve this, we relied on experts’ opinion to evaluate the indicators. Consequently, the indicators were presented at the 2021 Annual (Post-) Doctoral Colloquium of Land and Property Management Section of the Germany Geodetic Commission, which took place in November 2021 in Bonn. The indicators were also shared individually with scholars in Land Management, Urban Health, Global Health, and Geo-Health to review and critique for final selection. In terms of validity, we focused on face validity where we solicited views and critiques of experts to determine if the indicators reflect the content of the concepts in question. We also sought views on redundancy and the wording of indicators to avoid ambiguity and reduce sources of unreliability. The key feedback from this evaluation was that the wording of the indicators lacks measurability—the measuring data were not obvious from the initial framing of the indicators. The second feedback was the need to justify the basis of inclusion of indicators and how they improve existing indicators. This feedback was incorporated into the design of the indicators, by rephrasing the wording of the indicators to indicate presence or absence of an issue or phenomenon, and by introducing a land tenure basis column in the table of indicators to justify indicators, a column for measuring data sources, and a column for how the indicators are supplementing existing indicators. The revision of the indicators also resulted in the addition of 1 indicator and removal of 3 indicators which were considered redundant. The feedback mechanism with experts allowed the indicators to evolve into a more applicable set of indicators that can then be applied in an empirical setting. Consequently, in the next section, we present a framed a list of comprehensive indicators which can be used to measure the relationship between land tenure security and health.

## 5. Framing a Set of Indicators for Measuring Land Tenure Security and Health Nexus

Given the established theoretical, conceptual, and limited empirical connection of land tenure security and health, the next logical step was to develop a set of indicators that mediate and measure patterns of land tenure security and health. We identified aspects and indicators of land tenure security that can be linked to health outcomes. Four constructs (environmental justice, psychological security, social cohesion, and infrastructure access) which previous studies have suggested to result in positive health outcomes, but which derive from aspects of land tenure security, were identified as mediating variables, based on which a list of 46 (excluding 3 health measures) indicators were constructed (see Table 1).

From Table 1, indicators 1–15 help to assess the explanatory variable—a household’s tenure security status—and to determine whether one is tenure secure or insecure. The subsequent indicators help to measure each of the four mediating constructs that connect land tenure security to health. Together, indicators 16–23 are used to ascertain a household’s environmental justice status to the extent that these indicators are facilitated by tenure security and have health salience. Indicators 24–32 measures households’ levels of social cohesion on the premise that they are induced by tenure security and have health relevance. Similarly, indicators 33–40 assesses household infrastructure access to the effect that infrastructure access is a known predictor of health outcomes. Finally, indicators 41–46 help measure psychological security to the extent that it emanates from secure tenure and promotes health.

To explore the associations between land tenure security and health outcomes, we identified three health measures for ascertaining a respondent’s health status (indicators 47–49): self-rated health, number of visits to the hospital for self-treatment in the past year, and clinically diagnosed diseases in the past. The self-rated health, number of visits to the hospital, and past diagnosed disease are different health measures against which subsequent analysis will test their relationship with land tenure security and whether there exists a gradient between people exposed to different levels of land tenure security across the different health measures.

Indicators 1–46 each have equal weights to the extent that they have a range of zero to one— a score of zero where collected data on the indicator contributes negatively to the construct it measures and a score of one where collected data on an indicator contributes positively to the construct it measures. Below, we illustrate the derivation of the composite index for each measured construct at neighborhood level and how the relation between land tenure security and health will be estimated.

i. For all indicators of land tenure security (Indicator1…Indicator15), if response on an indicator contributes positively to land tenure security, then assign a score of 1 to the indicator (otherwise assign a score of 0).
ii. Land tenure security score for each household unit is (*T_h_*) = ∑ (Indicator1…indicator15).
iii. Land tenure security composite index for each neighborhood (*T_NB_*) is:
  $$T_{NB}=\frac{\sum_{h=1}^{n}T_{h}}{n}$$
iv. For all tenure-enabled indicators of environmental justice (Indicator16…Indicator23), if response on an indicator contributes positively to environmental justice, then assign a score of 1 to the indicator (otherwise assign a score of 0).
v. Environmental justice score for each household unit is (*E_h_*) = ∑ (Indicator16…indicator23).
vi. Composite index of environmental justice for each neighborhood (*E_NB_*) is:
  $$E_{NB}=\frac{\sum_{h=1}^{n}E_{h}}{n}$$
vii. For all tenure-enabled indicators of social cohesion (Indicator24…Indicator32), if response on an indicator contributes positively to land tenure security, then assign a score of 1 to the indicator (otherwise assign a score of 0).
viii. Social cohesion score for each household unit is (*S_h_*) = ∑ (Indicator24…indicator32).
ix. Composite index of social cohesion for each neighborhood (*S_NB_*) is:
  $$S_{NB}=\frac{\sum_{h=1}^{n}S_{h}}{n}$$
x. For all tenure-enabled indicators of infrastructure access (Indicator33…Indicator40), if response on an indicator contributes positively to land tenure security, then assign a score of 1 to the indicator (otherwise assign a score of 0).
xi. Infrastructure access score for each household unit is (*I_h_*) = ∑ (Indicator33…indicator40).
xii. Composite index of infrastructure access for each neighborhood is:
  $$I_{NB}=\frac{\sum_{h=1}^{n}I_{h}}{n}$$
xiii. For all tenure-enabled indicators of psychological security (Indicator41…Indicator46), if response on an indicator contributes positively to land tenure security, then assign a score of 1 to the indicator (otherwise assign a score of 0).
xiv. Tenure security score for each household unit is (*P_h_*) = ∑ (Indicator41…indicator46).
xv. Composite index for psychological security for each neighborhood is:
  $$P_{NB}=\frac{\sum_{h=1}^{n}P_{h}}{n}$$

**Figure 3:**
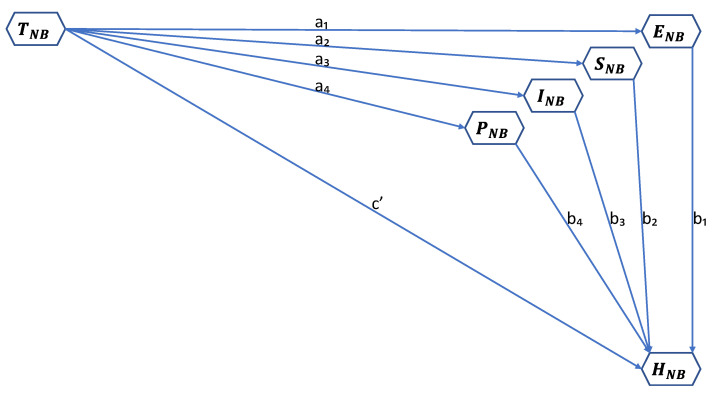
Structured equation model of the relation between land tenure security and health.

Consequently, the crux of analysis would lie in using structured equation modelling and mediation analysis to determine how composite index scores of land tenure security correlate with composite index scores of environmental justice, psychological security, social cohesion, and infrastructure access, and thus to health outcomes. Figure 3 illustrates the general structured equation model for measuring the relation between land tenure security and health. We adapted Ditlevsen et al.’s [107] mediation proportion approach for estimating exposure effect on an outcome explained by an intermediate variable. The mediation proportion is the measure of the part of an exposure effect on the outcome, explained by a third, intermediate variable(s). By this approach, the relation between land tenure security and health would be the total indirect effect of the explanatory variable (land tenure security) to its total effect on the outcome variable (health) through the mediating variables (environmental justice, psychological security, infrastructure access, and social cohesion). This approach has been applied by other scholars [107,108] in randomized clinical trials of the effects of interferon-α on visual acuity in patients with age-related macular degeneration, as well as to find the proportion of a social class effect on a health outcome that is mediated by psychologic variables. Therefore, we applied its logic to measure the relation between land tenure security and health.

From Figure 3, the relation between land tenure security and health can be estimated with the following equation:

$$TH_{NB}=\frac{a_{1}b_{1}+a_{2}b_{2}+a_{3}b_{3}+a_{4}b_{4}}{c\prime +a_{1}b_{1}+a_{2}b_{2}+a_{3}b_{3}+a_{4}b_{4}}$$

where $TH_{NB}$ is land tenure security and health nexus of neighborhood, $H_{NB}$ is health outcome of neighborhood, and c′, $a_{1}\ldots a_{4}$ and $b_{1}\ldots b_{4}$ are the path co-efficient of the respective variables.

## 6. Discussion and Conclusions

Table 1 is a key novel result, as it addresses the need and potentiality to tap into the synergies between land tenure security indicators and health indicators to promote global urban health. We note from reviewing existing frameworks of urban indicators [86], such as the Urban Livability Index, Public Health Indicators, Urban Health Indicators (Index), and Demographic Health Surveys, that although they refer to health factors such as housing and access to space, they bundle land tenure security as a single indicator of urban health as though land tenure security is an absolute concept. The indicators presented in this study are relative both in terms of the understanding of land tenure security and the context in which the indicators are applicable. Primarily, the indicators derive from the assumption that land titles alone are not a true measure of land tenure security and as such are particularly suited for the context of African countries, where the plurality of land tenure gives rise to differing understandings and multiple sources of land tenure security. Therefore, these indicators may not entirely be applicable in the Global North, where land tenure security is viewed dominantly from a legal lens and within simple tenure categories of possession of land title versus non-possession of title and homeownership versus rental.

Previous studies [16,18] have alleged the relationship between health and land tenure, but failed to demonstrate pathways of the link and empirically investigate the relationship. We demonstrated conceptually the pathways of association between land tenure security and health in an earlier paper [15]. In this paper, we showed how we could measure the paths of association between land tenure security and health, based on which could go further to investigate if there is an empirical relationship based on survey data we would be collecting.

The advantage of the indicators in Table 1 is that they target intra-urban and small-area level data for addressing health disparities, especially between informal and peri-urban areas and the rest of the city. Often, routinely available data on health and land tenure (security) can only be found at the national level, which masks the disparities that exist within urban areas. These indicators are useful for both comparative and evaluative studies within urban areas, especially in urban renewal and slum upgrading projects. They can be applied as pre-tenure or post-tenure regularization situations to compare and ascertain the health impact of land tenure regularization and urban resettlement projects.

Indicators are generally limited in that they can be driven by judgements about selecting and highlighting certain data over others [14]. Yet, these judgments do not render indicators unscientific or invalid, instead they are open to interpretation and re-evaluation. We have learned from our approach that designing indicators for fluid concepts such as health and land tenure security can be a daunting task as the two concepts do not mean the same thing for all persons. In addition, while it is a reality that land tenure security and health intersect in different aspects in our everyday living, the mediated nature of the link between the two concepts makes it largely driven by inferences and leaves room for expert bias. Nonetheless, such cross-concept inferences are needed for addressing social phenomena such as health in cities.

From our validation approach, we learned that the social, perceptive, and psychological nature of land tenure security presents a measurement challenge and that indicators may be relevant for understanding its dynamics, but they are extremely difficult to measure or combine with existing quantitative measures in health domains. We note as a limitation of our validation approach that by collecting opinions provided by experts individually, our approach is bereft quantitatively of each expert using numerical scales to rank each of the indicators. Additionally, we would have liked to include a few respondents from our study area in the validation process to make the validation more complete, but this was not possible. Nevertheless, the qualitative assessments, comments, and reasoned opinions of six experts from Land Management, Geo-Health, and Global-Urban Health domains, who were knowledgeable in the subject under investigation, were enough to identify the strengths and weaknesses of the indicators in terms of validity and reliability, based on which the indicators were revised, reworded, and some deleted.

The novelty of the indicators designed in this study lies in singling out land tenure security from the plethora of urban health indicators and unravelling the nuances of tenure security that affect health. The indicators leverage social relations, rights, and psychological aspects of land tenure security to define health links—an approach that deviates from traditional land tenure security indicators formulation where focus is put on economic benefits and investment or production incentives that derive from land tenure security. We have proposed 33 additional indicators that supplement existing indicators of land tenure security to measure health outcomes (see Table 1 for specific supplementary indicators). Although these indicators do not claim to capture the full complexity of land tenure security, they reflect land tenure security as a social determinant of health, which is important for global urban health and well-being.

This work provides an example of collaboration across land management and global urban health literature and proposes a set of indicators for measuring land tenure security and health relationships. Consequently, the frontiers of land tenure security and its components have been extended in this study to carry health meaning while broadening the scope of social determinants of health to include tenure security dimensions. The proposed indicators have the potential to support assessments and health profiling of urban neighborhoods and provide useful information to global urban health experts and decision makers to identify patterns in health outcomes for interventions. The indicators are also useful for intra-city profiling of neighborhoods according to their land tenure security scores and taking steps to make land tenure security interventions where necessary. Relating land tenure security to health is indispensable, policy-wise, in the management of urban areas to achieve Sustainable Development Goals (SDGs). It is well recognized that secure tenure is foundational and linked to several SDGs, including inclusive, safe, and resilient cities, poverty alleviation, eradication of hunger, gender equality, and productive land use, all of which have health salience.

There is the need for new frontiers of knowledge that continually identify pathways of associations between social phenomena and health outcomes, as well as indicators to measure and compare health variabilities and inequalities among urban populations. Policy discourses on urban health hardly concern land tenure security, and the collection of health determinants and indicators often gloss over land tenure security as a key predictor of health. We have suggested in this study that land tenure security through several identified indicators can interact with other mediating factors to modify health outcomes. The study does not suggest that land tenure security is the panacea to all health issues in urban areas. Rather, the study calls attention to the critical paths of connection of land tenure security with health outcomes and proposes indicators that can be used to measure and compare land tenure security and health relationships under different land tenure situations and settings.

In closing, we want to acknowledge this research’s weakness (or limitation) amidst several achievements. The study has strength because it achieved five very crucial goals. First, it used existing literature to argue that land tenure security, as a condition, serves as preventive medicine for improved health. Second, it identified an essential gap in land tenure security and health nexus and filled that gap. This gap has been neglected in previous research within the land management discipline [6,109]. Third, it conceptualised an evaluative framework for measuring land tenure security and health. Fourth, it then identified (or framed) a set of measuring indicators for land tenure security and health nexus. Lastly (and fifth), it illustrated how the composite indicator construction technique can be applied in measuring or estimating the relation between land tenure security and health. The study shows that it is possible to create a holistic framework of indicators that have both land tenure security and health salience. The designed indicators are appropriate and significant for testing the relation between land tenure security and health in the sense that they derive from established frameworks of tenure security and urban health indicators that have been previously tested. Inputs and feedback from experts in both land management and health domains also lend credence to the appropriateness and applicability of the designed indicators. However, the research (similar to many other scientific works) has its share of weaknesses (or limitations). A fundamental weakness of the study is that the composite indicator construction technique (used to illustrate how the relationship between land tenure security and health can be estimated) has not been tested with empirical data that can lead to the understanding of its actual application for drawing solid conclusions capable of being applied in human settlements. It is hoped that, based on the goals achieved, the next stage of research would be actual testing of the index in real-time.

The application of these indicators to a study area in Ghana and the extent to which these indicators measure the hypothesized relations between land tenure security and health will be the subject of a subsequent investigation and publication. The source of data for such an empirical study would be through geo-survey questionnaires. We find this a better option due to a lack of comprehensive databases that capture our variables of interest, and where they exist, are not available at the neighborhood scale. We envisage a sample size of 300–500 households to conduct an empirical study, with the main hypothesis being that variation in land tenure security leads to (in)direct variations in health outcomes, where people with tenure insecurity are likely to report poor health outcomes. This study is only the beginning of a new research dimension that introduces land tenure dynamics into global urban health discourse. Future research should explore how tenure security dynamics can modify the health effects of urban renewal, tenure regularization, slum upgrades, or urban greening projects. Further empirical research is needed to make health an explicit objective in tenure intervention projects and tenure security an explicit parameter in health impact assessments. Therefore, exploring the application of the tenure-health nexus composite index proposed in this study would allow for a better understanding of how urban land tenure policy decisions impact health and well-being empirically.

## Figures and Tables

**Table 1:** Set of measuring indicators for land tenure security and health nexus.

No	Indicator	Tenure Basis of Indicator	Measuring Data	Measured Construct	Connection to Health	Supplementing Existing Indicators of Land Tenure Security	Supporting Sources
1.	Presence of legally recognized proof of tenure and rights to land	Provability	Proportion of households with title, deed, or other legal proof of land ownership	Land tenure security	Enables four known predictors of health: social cohesion, psychological security, environmental justice, and infrastructure access.	No	[15,20,21,22,24,28,31,33,56,68,74,76,78,80,81,82,83,84,85,86]
2.	Presence of other documentation other than legally recognized proof of tenure and rights to land	Provability	Proportion of households with other documents to land other than legal proof of ownership			No	
3.	Occupation of land for a minimum of 12 years in accordance with Limitation Act	Periodicity	Proportion of households who feel qualified to invoke Limitation Act to claim possession of land			Yes	
4.	Presence of municipal infrastructure and utility services	Recognition	Proportion of households with formal connection to water, electricity, and sewage systems			Yes	
5.	Access to political power and people of influence	Assurance	Proportion of households who think they cannot lose their land due to their strong political affiliation and access to influential people			Yes	
6.	Presence of evidence recognition of land rights by government, local community, and institutions	Legitimacy	Proportion of households who view their land rights as legitimized by government and local institutions such as district assemblies, and property and utility rate collectors			No	
7.	Presence of experience of actual (threat of) eviction or dispossession	Periodicity	Proportion of households who have received threats or notice of eviction in the past			No	
8.	Presence of experience of actual loss of tenure and land rights in the past	Periodicity	Proportion households who have either been evicted from their homes or lost their land in the past			No	
9.	Presence of perceived risk of future loss of tenure and land rights	Periodicity	Proportion of households who fear they are likely to lose their land or homes in the future			No	
10.	Presence of state protection against dispossession or eviction	Assurance	Proportion of households with confidence in the state to protect their land rights against arbitrary loss			No	
11.	Likelihood of losing tenure and land rights to natural disasters like floods and fire outbreaks	Periodicity	Proportion of households who report experiencing episodes of flood or fire and fear losing their land or homes to these natural disasters			Yes	
12.	Presence of autonomy over bundle of land rights held	Alienability and exclusivity	Proportion of households either agree or strongly agree they can sell, lease, sub-divide, collateralize, develop, or use their land or house without restrictions			Yes	
13.	Presence of experience of land use or boundary disputes	Legitimacy and periodicity	Proportion of households who have experienced land use or boundary conflict in the past				
14.	Nearness to land use or boundary dispute in the past	Legitimacy and periodicity	Proportion of households in the neighborhood who live within 1 km of a known or reported land use or boundary dispute			No	
15.	Presence of approved land use plan	Space and legitimacy	Proportion of households whose land or houses are situated on or demarcated by an approved land use plan			Yes	
16.	Exercise of right to legal redress for environmental ills due to secure tenure	Rights	Proportion of households who report using legal means to seek justice for environmental pollution they suffered from others	Environmental justice	Land tenure security activate environmental rights, responsibilities, and restrictions which allow individuals and communities to take transformative actions for environmental goods and against environmental ills to promote environmental health	Yes	[15,87,88,89,90,91,92,93,94,95,96,97]
17.	Exercise of responsibility towards waste disposal and keeping environments clean due to secure tenure	Restrictions and responsibility	Proportion households who think environmental littering is a problem and use designated waste collection points and methods to dispose waste			Yes	
18.	Exercise of right to restrict others from polluting land and environment due to secure tenure	Exclusivity	Proportion of households who report coercing others to dispose waste correctly and prevent others from littering their land and environment			Yes	
19.	Exercise of right to defend land and environment without threats or fear of harassment due to secure tenure	Rights	Proportion of households who either agree or strongly agree they can freely defend their land and environments against environmental injustice			Yes	
20.	Nearness to solid waste disposal site due to insecure tenure	Livability	Proportion of households who live within 500 m of a waste disposal site			Yes	
21.	Access to good environmental quality and amenities such as urban green and blue spaces	Benefits and privileges	Proportion of households within 800 m walking distance of a park			Yes	
22.	Access to and participation in environmental policy and decision making due to secure tenure	Benefits and privileges	Proportion of households who participated in citizen engagement activities relating to environmental policy and decision making in the last year			Yes	
23.	Exercise of right to protest and activism for safe environments due to secure tenure	Rights and livability	Proportion of households who participated in environmental protest and activism in the last year			Yes	
24.	Feeling of sense of belonging due to duration of tenure	Periodicity and recognition	Proportion of households who either agree or strongly agree with feeling they belong to their neighborhood	Social cohesion	Constancy afforded by secure tenure promotes social health by leveraging on sustainable social ties and networks	Yes	[15,86,88,98,99,100]
25.	Presence of sustained friendships and social relationships due to residential stability	Periodicity and recognition	Proportion of households who either agree or strongly agree they have built lasting social ties and friendship with their neighbors due to length of stay			Yes	
26.	Feeling of sense of community	Periodicity	Proportion of households who either strongly agree or agree they feel they are part of the community			Yes	
27.	Participation in communal activities and advocacy	Periodicity	Proportion of households who report they participate in organized community activities such as clean-up campaigns, protests, and communal labor			Yes	
28.	Sense of security, connectedness, and trust	Livability	Proportion of households who either agree or strongly agree with the belief that their neighbors would help them in an emergency			Yes	
29.	Presence of experience of social conflict within community	Recognition	Proportion of households who report ever experiencing discrimination, been prevented from using land or doing something, or been hassled or made to feel inferior because of their race, ethnicity, or color			Yes	
30.	Feeling of attachment to place due to duration of tenure	Periodicity	Proportion of households either agree or strongly agree they do not want to relocate because they have become attached to their neighborhood			Yes	
31.	Sense of inclusion and reduced segregation and exclusion from right to the city and benefits of city life	Recognition	Proportion of households in the neighborhood who agree they have equal opportunities to inhabit, use, participate, and influence decisions pertaining to urban space			Yes	
32.	Presence of social support, social capital, and empowerment	Livability	Proportion of households in the neighborhood who feel they can count on neighbors			Yes	
33.	Access to municipal water supply	Livability	Proportion of households served by municipal water supply system, borehole, or tanker service	Infrastructure access	Tenure security provides legitimacy and entitlement to state-provided infrastructure, or incentive for private investment in life sustaining infrastructure that promote physical health	Yes	[15,19,56,63,64,86,101]
34.	Access to municipal waste collection services	Livability	Proportion of households served by municipal or private waste collection service providers			Yes	
35.	Access to municipal sewer infrastructure	Livability	Proportion of households who are served by municipal solid waste management system			Yes	
36.	Access to waste or garbage collection facility	Livability	Proportion of households within 75 m walking distance of a designated wastebin, who are connected to a wastewater collection or treatment facility			Yes	
37.	Access to privately installed toilet facility	Livability	Proportion of households served by a privately installed toilet			Yes	
38.	Access to adequate shelter	Space and Livability	Proportion of households living in durable structures (as per the SDG-era definition of housing)			No	
39.	Presence of private capital investment in housing improvement	Livability	Proportion of households who have made capital improvements on the properties they own in the past 12 years				
40.	Willingness of state to provide utility services and social infrastructure	Recognition and legitimacy	Proportion of households who report ease of getting local authorities to provide social infrastructure or utility services				
41.	Feeling of control and ability to exercise autonomy of decisions over land or property	Exclusivity	Proportion of households who either agree or strongly agree they can exercise autonomy in decisions pertaining use of their land	Psychological security	Reduced psychosocial stresses and anxieties associated with insecure tenure and eviction threats promote mental health.	Yes	[15,54,102,103,104,105]
42.	Absence of anxiety and fear of losing tenure and land rights	Periodicity	Proportion of households who either disagree or strongly disagree that they have anxiety and fears of losing their land or homes			No	
43.	Feeling of prestige and high self-esteem due to secure tenure status	Recognition	Proportion of household who rate their sense of pride and self-esteem in their homes and living conditions as high or very high			Yes	
44.	Feeling of safety and privacy due access to delineated space	Space	Proportion of households who report feeling safe and having privacy in their homes			Yes	
45.	Sense of routine due to constancy of tenure	Periodicity	Proportion of households who feel they have a home around which their daily life activities are constructed			Yes	
46.	Existence of antagonistic relationship with government and state institutions	Recognition	Proportion of households who report being under constant threat by state institutions over legitimacy of where they live			Yes	
47.	Self-rated heath	N/A	Proportion of households who either rate their health as good or very good	Health		No	[54,106]
48.	Number of visits to the hospital for self-treatment in the past year	N/A	Number of times a household (member) visited the hospital in the past year			No	
49.	Clinically diagnosed diseases in the past year	N/A	Proportion of households who report being diagnosed with specific diseases in the past year			No	

## Data Availability

Not applicable.
